# Stem-like T cells in cancer immunotherapy: biology, regulation and therapeutic targeting

**DOI:** 10.3389/fimmu.2026.1764549

**Published:** 2026-02-25

**Authors:** Hui Wang, Zhuoran Yao, Ren Luo, Kai Kang, Feifei Na, You Lu

**Affiliations:** 1 Division of Thoracic Tumor Multimodality Treatment, Cancer Center, West China Hospital, Sichuan University, Chengdu, Sichuan, China; 2 Department of Radiation Oncology, Cancer Center, West China Hospital, Sichuan University, Chengdu, Sichuan, China

**Keywords:** cancer immunotherapy, self-renewability, stem-like T cells, therapeutic implications, tumor microenvironment

## Abstract

The identification of stem-like CD8^+^ T cells, also termed progenitor or precursor of exhausted T cells (T_PEX_), has reshaped our understanding of durable antitumor immunity. These cells exhibit progenitor-like properties, including self-renewal capacity and multilineage differentiation potential, giving rise to both effector-like and terminally exhausted CD8^+^ T cell subsets. Accordingly, the abundance of stem-like CD8^+^ T cells correlate strongly with improved clinical outcomes in patients receiving immune checkpoint inhibitors, adoptive cell therapy, or cancer vaccines across multiple tumor types. This review synthesizes recent advances in T_PEX_ cells biology, highlighting interconnected research pillars, including: specialized niche microenvironments that sustain stemness of T_PEX_ cells through coordinated chemokine signaling and antigen-presenting cell interactions; core molecular circuitry that dynamically balances self-renewal versus effector differentiation via transcription factors and cytokines; and therapeutic reprogramming strategies that harness T_PEX_ cells as the primary driver of immunotherapy efficacy. Further, we explore strategies to augment the functionality of T_PEX_ cells through niche modulation, stem-like CAR-T engineering, and combinatorial approaches, highlighting the trend that targeting T_PEX_ cells thus emerge as a transformative future strategy to overcome immunotherapy resistance and achieve a durable response.

## 1 Introduction

The advent of immunotherapy has revolutionized cancer treatment. However, durable responses remain limited, occurring in only 15-30% of patients receiving immune checkpoint blockade (ICB) (1, 2). Preclinical and clinical evidence underscores that CD8^+^ T cell infiltration correlates with improved outcomes, particularly in cancers with high mutational burden and neoantigen load (3). Critically, the functional outcomes of antigen-stimulated CD8^+^ T cells are critically shaped by the context and duration of antigen exposure.

During acute antigen stimulation (as in resolved infections or some vaccines), CD8^+^ T cells differentiate into both short-lived effector cells (SLECs) characterized by a KLRG1^+^ CD127^−^ phenotype and memory precursor effector cells (MPECs) characterized by a KLRG1^−^ CD127^+^ phenotype (4, 5). SLECs exhibit potent cytotoxicity and undergo robust clonal expansion to mediate immediate pathogen clearance, but most undergo apoptosis after antigen clearance. Whereas MPECs are minimally differentiated, activated CD8 T cells that show a high propensity to survive during the transition from an activated state to a resting state. Importantly, it is the MPECs population that gives rise to long-lived memory T cells (2, 4, 6–8). Additionally, MPECs afford long-lived protective immunity by virtue of their ability to generate large waves of effector cells in the face of renewed antigen stimulation; their ability to rapidly recall effector functions; and their broad distribution in peripheral tissues where they can act promptly to precipitate tissue immunity and memory T cells (2, 5).

**Figure 1:**
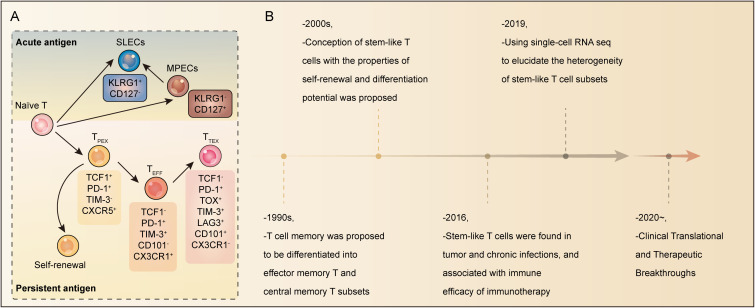
Historical and characteristics of stem-like T cells. **(A)** T cell heterogeneity and differentiation hierarchy in acute and chronic infection. (Up) Acute antigen stimulation drives CD8^+^ T cells differentiate into short-lived effector cells (SLECs) characterized by a KLRG1^+^ CD127^−^ and memory precursor effector cells (MPECs) characterized by a KLRG1^-^ CD127^+^ CD62L^+^ CD27^+^. When encountering the antigen again, MPECs will rapidly differentiate into SLECs. (Down) Persistent antigen stimulation drives CD8^+^ T cells into exhausted T cell subsets including progenitor of exhausted T cells (T_PEX_), effector-like exhausted T cells (T_EFF_), and terminal differentiated exhausted T (T_TEX_) cells. T_PEX_ cells express TCF1, PD-1, and CXCR5 continuously self-renew and replenished the T_PEX_ pool, thus giving rise to more differentiated TCF1^-^ PD-1^+^ TIM3^+^ CD101^-^ T_EFF_ cells and TCF1^-^ PD-1^+^ TIM3^+^ CD101^+^ T_TEX_ cells. **(B)** Timeline of historical milestone events in the field of stem-like T cells.

Conversely, chronic antigen stimulation, as occurs in persistent viral infections and cancer, drives CD8^+^ T cells into a state of exhaustion or dysfunction, which is distinct from functional memory (5, 9–12). Within this exhausted compartment, a distinct subset with stem-like or progenitor properties has been identified, variably termed progenitor/precursor of exhausted T (T_PEX_) cells or stem-like CD8^+^ T (T_SL_) cells (2, 13–16). This subset is defined by two cardinal stem cell-like functions: self-renewal, which maintains a durable reservoir, and multilineage differentiation potential, enabling them to give rise to both effector-like exhausted T (T_EEF_) cells and terminally differentiated exhausted T (T_TEX_) cells (9, 17–19) (**Figure 1A**). These progenitor-like properties of T_PEX_, sustained by a core transcriptional circuit such as transcription factor 1 (TCF1), allow them to serve as a renewable source for effector T cells and underpin durable immune responses, distinguishing them from terminal effector or classical memory subsets (4, 20–22). Notably, T_PEX_ cells are the primary mediator of the proliferative burst following ICB and are essential for sustained tumor control (14, 20, 23, 24). The study of T_PEX_ cells has evolved through key milestones (**Figure 1B**). They were first described in 2005 in the context of murine graft-versus-host disease (25). A pivotal advance came in 2016, when studies in chronic infection and tumor models revealed that a stem-like progenitor subset within the exhausted lineage drives T cell regeneration upon PD-1 blockade, providing a mechanistic basis for ICB efficacy (11, 26). The advent of single-cell RNA sequencing has since resolved the transcriptional heterogeneity of T_PEX_ cells within the tumor microenvironment (TME), uncovering finer regulators of their maintenance (17). Most recently, these insights have spurred clinical innovation, with stem-like CAR-T therapies showing improved persistence (27) and epigenetic reprogramming strategies aiming to rejuvenate stem-like functionality (28, 29).

Herein, we review the latest advances in understanding T_PEX_ cell biology within the TME, focusing on: (1) the specialized niches that support their maintenance and differentiation, (2) strategies to generate or enhance T_PEX_ cells, and (3) the therapeutic potential of targeting T_PEX_ functionality to improve ICB, adoptive cell therapy, and cancer vaccination.

## 2 Molecular markers and characteristics of stem-like T cells

### 2.1 Molecular markers of stem-like T cells

Stem-like T (T_SL_) cells, also known as precursors of exhausted T (T_PEX_) cells originate from antigen-stimulated naïve T cells. But under the chronic or persistent antigen exposure (as in cancer or chronic infections), they emerge as a distinct subset within the exhausted T cell lineage. Their canonical and defining feature is the high expression of the transcription factor TCF1, encoded by *Tcf7*, a key regulator of T cell stemness and a downstream effector of the Wnt/β-catenin pathway (22, 30–32). Phenotypically, these cells are characterized by the co-expression of TCF1 and intermediate-to-high levels of PD-1 (TCF1^+^ PD-1^+^), which distinguishes them from both naïve T cells (TCF1^+^ PD-1^−^) and T_TEX_ cells (TCF1^−^ PD-1^+^). They typically lack or express low levels of markers associated with terminal exhaustion, such as TIM-3. Additional surface markers often found on T_PEX_ cells include CXCR5 (important for lymphoid follicle homing) and *Slamf6* (Ly108 in mice) (33), while they exhibit low expression of immediate effector molecules like granzyme B (GZMB) and interferon-γ (IFN-γ) (5, 9, 34, 35). Details are summarized in **Table 1**.

**Table 1:** The definition, markers, function, and metabolic profile of T cell terminologies.

Stimulation	T cell lineage	Subset	Markers	Key feature	Metabolic profile	Reference
Acute antigen stimulation	Activated/memory T cells	SLEC	KLRG1^+^, CD127^-^	Highly differentiated cytotoxic CD8^+^ T cell; Most undergo apoptosis after antigen clearance.	Highly glycolytic and dependent on one-carbon metabolism	(4, 6–8, 38, 39)
		MPECs	CD127^+^, CD27^+^, TCF1^+^, CD62L^+/-^, KLRG1^-^	A minimally differentiated activated CD8^+^ T cell; Has a high propensity to survive during the transition from an activated state to a resting state; Produce cytokines but exhibit less cytotoxicity than SLECs.	Dependent on OXPHOS and mitochondrial function; Contain relatively greater mitochondrial mass; The mitochondria have a fused ultrastructure and a relatively higher SRC.	(4, 36–40)
Chronic antigen stimulation	Exhausted T cells	T_PEX_*	TCF1^+^, PD-1^+^, BCL6^+^, SLAMF6^+^, CXCR3^+^, LEF1^+^, CD73^+^, XCL1^+^, CXCR5^+^, TIM3^-^, CD39^-^, granzyme B^-^	Self-renewal; Expands and burst proliferate after ICB; Differentiate into T_EFF_ cells and T_TEX_ cells.	Mitochondrial fitness (high SRC, fused morphology); Increased FAO and mitochondrial SRC, generated less reactive oxygen species, and minimized oxidative damage.	(2, 4, 5, 9, 13–16, 23, 24, 39–42)
		T_INT_	PD-1^+^, TIM3^+^, T-bet^+^, granzyme B^+^, perforin^+^, IFNγ^+^, CX3CR1^+^, TCF1^-^, SLAMF6^-^, CD101^-^	Express effector molecules such as granzyme B and perforin to kill tumor cells.	Metabolic insufficiency and inhibition of mitochondrial respiration and glycolysis.	(2, 4, 5, 9, 17, 19, 39–42)
		T_TEX_	PD-1^+^, TOX^+^, TIM3^+^, granzyme B^+^, CD39^+^, CD101^+^, TCF1^-^, SLAMF6^-^, CX3CR1^-^	Increased expression of inhibitory receptors; Limited killing capacity and proliferation.	Severe mitochondrial dysfunction driven by PGC1α suppression; Metabolic paralysis, such as decreased glycolytic activity and OXPHOS.	(2, 4, 5, 9, 17, 19, 39–42)

T_PEX_, also named T_SL_;

T_INT_, also named T_EFF_.

SLECs, Short-lived effector cell; MPECs, Memory precursor effector cells; T_PEX_, Progenitor or precursor exhausted T; T_SL_, Stem-like T; T_INT_, Intermediate exhausted T; T_EFF_, Effector-like exhausted T; T_TEX_, Terminally differentiated exhausted T; SRS, Spare respiratory capacity; FAO, Fatty acid oxidation; ICB, Immune checkpoint blockade; OXPHOS, Oxidative phosphorylation.

### 2.2 Functional characteristics of stem-like T cells

Functionally, T_PEX_ cells are demarcated from other T cell subsets by their unique combination of properties. Unlike naïve T cells, which are antigen-inexperienced, T_PEX_ cells are generated post-activation and possess a poised, antigen-experienced state while retaining a multipotent capacity. Compared to SLECs, which are terminally differentiated for immediate cytotoxicity but undergo rapid contraction. T_PEX_ cells exhibit minimal immediate effector function but sustain long-term proliferative potential and self-renewal. They also differ from conventional memory T cells (e.g., central memory and effector memory T cells), which arise from acute, resolved infections and are maintained in a quiescent state for rapid recall (4, 36, 37).

T_PEX_ cells exist within the context of persistent antigen, are often part of the “exhausted” lineage, and their self-renewal is continuously engaged to replenish exhausted effector pools. Most critically, they are distinct from T_TEX_, which are epigenetically fixed, dysfunctional, and possess negligible proliferative capacity (28). T_PEX_ cells serve as the primary reservoir that undergoes proliferative expansion in response to ICB, driving the replenishment of the effector T cell compartment, which is absent in T_TEX_ subsets (2, 14). Overall, these functional profiles including self-renewal under chronic antigen pressure, multilineage differentiation, and therapy-responsive proliferation, defines their unique role as the central regenerative engine of the antitumor T cell response. Details are summarized in **Table 1**.

### 2.3 Metabolic signature of stem-like T cells

This metabolic profile of T_PEX_ cells starkly contrasts with other T cells. SLECs are highly glycolytic and dependent on one-carbon metabolism (38, 39). Memory T cells contain relatively greater mitochondrial mass, and the mitochondria have a fused ultrastructure and a relatively higher spare respiratory capacity (SRC) (38–40). T_TEX_ cells exhibit severe mitochondrial dysfunction driven by PGC1α suppression and metabolic paralysis, such as decreased glycolytic activity and oxidative phosphorylation (OXPHOS). Whereas T_PEX_ cells display distinct metabolic profiles characterized by increased fatty acid oxidation (FAO) and mitochondrial SRC, which generates less reactive oxygen species, minimizing oxidative damage. Furthermore, they possess abundant, fused mitochondria, indicative of metabolic fitness. The unique metabolic wiring of T_PEX_ cells is thus integral to their self-renewal capacity, persistence, and readiness to proliferate upon demand (39, 41, 42).

## 3 Specific niches of stem-like T cells

When chronic antigen stimulation, T_PEX_ cells are activated and predominantly localize to specialized niches within tissues. Within these niches, they engage in intercellular interactions and receive microenvironmental signals critical for their survival and functional maintenance. These niches enable T_PEX_ cells to contribute to immune surveillance and mount rapid recall responses upon antigen re-encounter (**Table 2**). Consequently, delineating the specific niches harboring T_PEX_ cells is critical for advancing immunotherapeutic strategies and developing more efficacious treatments.

**Table 2:** Specific niches of stem-like T cells.

Specific niches	Key cell type	Function	Reference
TDLNs	DCs	Carry tumor antigens, activate T_PEX_ cells, and maintain their stem cell properties.	(44, 45)
	Fibroblast	Promotes the localization of T_PEX_ cells and stem cell phenotype through the CCR7-CCL19/CCL21 signaling pathway.	(43)
Perivascular tumor niches	APCs	Antigens are presented through MHC II molecules, which promote the aggregation and function of T_PEX_ cells.	(15, 49)
	Endothelial cells (CD31^+^)	Support T_PEX_ cells residency through the CXCR6-CXCL16 and CXCR3-CXCL9/CXCL10 signaling pathways.	(22, 50)
TLS	B cells	Promote the aggregation and function of T_PEX_ cells through CXCL13 signaling, and support anti-tumor immune responses.	(34, 53, 54)
	Interaction between DCs and T cells	In TLS, DCs interact with T_PEX_ cells to maintain their stem cell properties and functions.	(34, 52)

TDLN, Tumor-draining lymph nodes; TLS, Tertiary lymphoid structures; DCs, Dendritic cells; APCs, Antigen-presenting cells; T_PEX_, Progenitor or precursor exhausted T.

### 3.1 Tumor-draining lymph nodes

**Figure 2:**
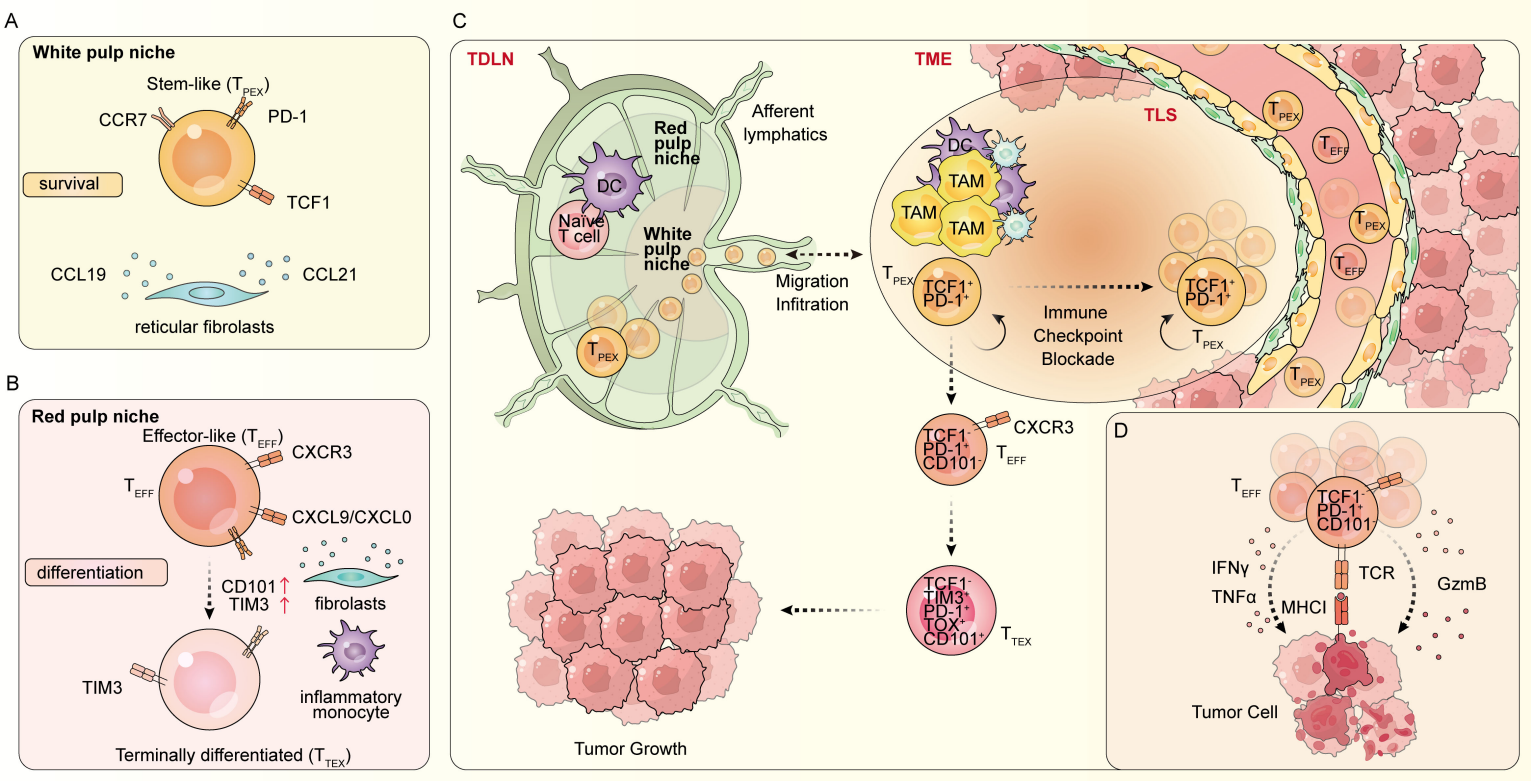
Special niches and differentiation of stem-like T cells. Progenitor of exhausted T (T_PEX_) cells reside in tumor-draining nodes (TDLNs) or lymphoid structures such as APC-niche and tertiary lymphoid structures (TLS) within tumors. Here, they interact with antigen-presenting cells (APCs), such as dendritic cells (DC) and macrophages, CD4^+^ T cells, and B cells that provide signals for T_PEX_ cell survival and maintenance and their differentiation into effector-like exhausted T cells (T_EFF_), and terminal differentiated exhausted T (T_TEX_) cells. **(A)** T_PEX_ cells migrate and localize in the white pulp niche (closer to the capsule and afferent lymphatics) of TDLN is regulated by the chemokine receptor CCR7. **(B)** T_EFF_ cells expressing CXCR3 are attracted to the red pulp niche (closer to the efferent lymphatics) by CXCL9 and CXCL10, produced by DCs and stromal cells and then differentiating into T_TEX_ cells. **(C)** Mature DCs migrate to TDLNs under the guidance of chemokines like CCL19 and CCL21 produced by fibroblast. Within TDLNs, DCs deliver tumor-derived antigens to naïve T cells via major histocompatibility complex (MHC) molecules, supported by co-stimulatory signals like CD80/CD86 binding to CD28. This antigen presentation primes naïve T cells, promoting their differentiation into T_PEX_ cells, which then continuously migrates from the TDLNs to the tumor and differentiated into T_EFF_ cells and T_TEX_ cells. **(D)** T_EFF_ cells expressing CXCR3 are recruited to both primary and abscopal tumor sites through the bloodstream, which is facilitated by chemokines such as CXCL9 and CXCL10. T_EFF_ cells secret IFNγ, TNFα, and Gzmb to kill tumor cells.

In patients with lung adenocarcinoma, *Connolly* et al. (36) observed that the majority of T_PEX_ cells were present in non-metastatic lung-draining lymph nodes, which was in line with the findings in mice, where T_PEX_ cells were predominantly located in tumor-draining lymph nodes (TDLNs). T_PEX_ cells within the TDLNs present with high CCR7 expression, which is vital for the migration and positioning of T_PEX_ cells. The stromal cells in TDLNs could produce CCL19 and CCL21, which are the ligands of CCR7, and attract T_PEX_ cells to concentrate in the inner T cell zone (TCZ) (43) (**Figure 2A**). Mature dendritic cells (DCs) carrying tumor-generated antigens infiltrate the TCZ, providing specific signals that tune naïve T cells toward T_PEX_ cells (44, 45). Meanwhile, in the outer TCZ, DCs attract T_PEX_ cells to differentiate into effector cells by expressing CXCR3 ligands, CXCL9 and CXCL10, and IFN-I (**Figure 2B**). Thus, blockading of PDL1 on DCs could induce local expansion of T_PEX_ cells within the TDLNs, which further traffics to the tumor and induces effective immunity (16, 46, 47). Other studies also discovered that T_PEX_ cells in the TDLNs are the precursors of the tumor-specific T cells (37, 48) and play a vital role in maintaining persistent T cell responses (**Figure 2C**). Altogether, increasing data have shown that TDLNs as a reservoir of T_PEX_ cells are key sites where *de novo* antitumor responses are initiated.

### 3.2 Perivascular tumor niches

T_PEX_ cells predominantly localize to the specialized perivascular niches at the tumor-stroma interface, while T_EFF_ cells and T_TEX_ cells infiltrate deeper tumor parenchymal regions, where they directly engage tumor cells. These perivascular niches provide specific signals that sustain T_PEX_ cell survival and stemness. Multiple studies have revealed a spatial correlation between T_PEX_ cells and antigen-presenting cells (APCs) in tumor tissues. In kidney, bladder, and prostate cancers, T_PEX_ cells localize preferentially to tumor regions densely populated by MHCII^+^ APCs, while T_TEX_ cells and T_TEX_ cells resided distally. This compartmentalization establishes functional perivascular niches that drive T_PEX_ cell clustering and facilitate cross-presentation of tumor antigens (15, 49).

Furthermore, the presence of perivascular niches correlated with enhanced tumor vascularization. In a melanoma, T_PEX_ cells are localized preferentially within perivascular regions adjacent to CD31^+^ endothelial cells in tumor tissue (22). Similar perivascular niches, enriched in T_PEX_ cells and DCs, have also been documented in colon cancer and pancreatic ductal adenocarcinoma (50). These niches are coordinated by CXCR6-CXCL16 and CXCR3-CXCL9/CXCL10 chemokine interactions within the tumor stroma, interactions critical for mediating immunotherapy responses. Crucially, immunotherapy induces the formation of these perivascular niches, and their abundance correlates with the magnitude of therapeutic response, supporting their functional importance in antitumor immunity (50).

### 3.3 Tertiary lymphoid structures

Tertiary lymphoid structures (TLS) are ectopic immune cell aggregates exhibiting architectural parallels to the follicles of secondary lymphoid organs (51). These structures facilitate antigen presentation to lymphocytes, thereby supporting the initiation and regulation of adaptive immune responses. Within the TME, TLS is predominantly localized at peri-tumoral sites or along the tumor-stroma interface (52) (**Figure 2C**). Their presence correlates positively with enhanced intratumoral T cell infiltration and favorable patient prognosis (34, 53). In the context of patients with stage I–IV non-small-cell lung cancer (NSCLC), T_PEX_ cells are observed to be located in the TLSs rather than in the tumor parenchyma (54). Studies further demonstrate significantly greater T_PEX_ abundance in TLS-enriched tumors, suggesting TLS may function as an intratumoral reservoir for T_PEX_ cells (34). Conversely, T cells from TLS-deficient tumors exhibit pronounced exhaustion phenotypes, characterized by elevated co-expression of PD-1 and TIM-3 (52). While burgeoning evidence underscores the prognostic and immunological significance of TLS and associated T_PEX_ populations, the precise molecular mechanisms governing their spatial organization, functional interplay, and therapeutic contributions remain incompletely elucidated.

## 4 Differentiation and maintenance of stem-like T cells

The differentiation and maintenance of T_PEX_ cells are governed by complex cell-extrinsic and -intrinsic factors. While sustaining self-renewal ability, T_PEX_ cells simultaneously undergo substantial transcriptional and functional reprogramming and phenotypic differentiation following antigen exposure (49, 55). Studies on chronic viral infections and tumor models have demonstrated that when T_PEX_ cells proliferate in response to persisting antigen or inflammatory cues, they may give rise to multiple exhausted progenies, including T_EEF_ cells, intermediate exhausted T (T_INT_) cells, and T_TEX_.

T_EEF_ and T_INT_ cells have been used to describe transitional exhausted T (transitional T_EX_) cells. These cells express effector molecules such as GZMB and perforin and have anti-viral and anti-tumor functions. They leave lymphoid tissues and migrate to sites of infection or tumors (56) (**Figure 2D**). Concomitant with this differentiation, transitional T_EX_ cells downregulate TCF1 expression, thereby losing the stem-like characteristics. Markers used for transitional T_EX_ cells are TCF1^−^ PD-1^+^ TOX^+^ TIM3^+^ CD101^−^ (4, 16, 30).

T_TEX_ cells have little proliferative capacity and reduced and altered effector function compared to transitional T_EX_ cells. T_TEX_ cells do retain limited cytotoxicity, produce low amounts of effector cytokines, and express chemokines that help recruit other leukocytes. T_TEX_ cells can arise directly from T_PEX_ cells and also from transitional T_EX_ cells. In the context of cancer, T_TEX_ cells are often referred to as ‘dysfunctional’ T cells. Markers used for T_TEX_ cells are TCF1^−^ PD-1^+^ TOX^+^ TIM3^+^ CD101^+^ (2, 9, 56). Collectively, the differentiation of exhausted CD8^+^ T cells follow a hierarchical and progressive pathway under sustained antigen exposure (**Figure 2D**).

Functionally, while undergoing differentiation, T_PEX_ cells retain self-renewal capacity, a property critical for robust proliferative expansion that underpins durable clinical benefit following immunotherapy. Conversely, T_EFF_ and T_TEX_ populations exhibit limited survival and self-renewal potential (2, 9, 57). Although reversal of T cell exhaustion has traditionally been considered the primary mechanism of ICB efficacy, studies on T_PEX_ cells demonstrate that their defining feature, responsiveness to ICB-mediated expansion, represents the key driver of therapeutic benefit, rather than phenotypic reversion (21, 58, 59). Notably, the T_EFF_ and T_TEX_ phenotype predominates within the tumor-specific repertoire, implying that sustained antitumor immunity likely depends on an external T_SL_ population capable of generation and infiltration (14, 60–62).

T_PEX_ cells exhibit migratory capacity, trafficking between intratumoral perivascular niches or TLS and reservoir sites within TDLNs (16, 36, 37, 63–65). Preclinical evidence using the S1P1-agonist FTY720 to block T cell egress demonstrated that preventing T_PEX_ migration diminished tumor regression. This challenges the paradigm that anti-PD-1 therapy acts solely on intratumoral T cells and underscores the necessity of TDLNs for T_PEX_ maintenance (16, 64).

## 5 Endogenous frequency and microenvironmental regulation of stem-like T cells

The frequency of T_PEX_ cells is highly variable and context-dependent, typically representing ~5%-20% of tumor-infiltrating CD8^+^ T cell across different cancer types and patients (15, 36, 66, 67). The variation is dynamically regulated by the TME through several key mechanisms.

### 5.1 Special niches

The frequencies of T_PEX_ cells are positively correlated with the presence of immunologically active structures, including TLS, perivascular areas, and TDLNs (50–54). These niches provide critical survival signals (e.g., IL7 and IL-15) and intermittent antigen presentation, which sustain T_PEX_ cell clustering and prevent terminal differentiation (51, 52, 54). Clinically, niche-rich tumors exhibit higher T_PEX_ cell frequencies and improved patient outcomes (68, 69). The absence of these structures correlates with lower T_PEX_ frequencies and a more exhausted T cell landscape (50–54, 68, 69). The TDLN serves as a critical extratumoral reservoir, maintaining a higher frequency of T_PEX_ cells that can be recruited to the tumor (36, 67). T_PEX_ cells continuously traffic between the TDLN and tumor, a process required for replenishing the intratumoral pool (36, 70, 71). Using FTY720 to block this egress could reduce T_PEX_ cell frequency and antitumor immunity (36, 70, 72).

### 5.2 Metabolic and antigen pressure

The nutrient-depleted, hypoxic TME imposes metabolic stress that can also affect the T_PEX_ cells. A clear consensus indicates that T_PEX_ cells rely on oxidative metabolism (OXPHOS/FAO) for long-term persistence, unlike their glycolytic effector progeny (39, 40). Furthermore, chronic antigen exposure such as high levels of IFN-I (late phase), IL-2, and inflammatory signals, constantly depletes the T_PEX_ pool by driving differentiation, making their sustained frequency a balance between self-renewal and differentiation pressure (37, 73).

### 5.3 Impact on cancer progression and patient outcomes

Additionally, the regulatory mechanisms governing T_PEX_ cell frequency described above also directly dictate the balance between tumor immune control and disease progression. A robust and well-maintained T_PEX_ pool, supported by functional niches and balanced cytokine signals (51, 52, 54), establishes a state of continuous immunosurveillance. This enables the adaptive immune system to dynamically respond to tumor evolution by generating T_EFF_ cells continuously. In this context, the immune system can control tumors in a state of long-term equilibrium or even mediate tumor regression, a hallmark of effective immunotherapy. Clinically, this is reflected in the strong association between high T_PEX_ abundance, the presence of TLS, and favorable patient outcomes across multiple cancer types (68, 69).

Conversely, the breakdown of T_PEX_-supportive regulation is a pivotal event driving cancer progression. This failure can occur through several interconnected mechanisms: 1) Loss of supportive niches (e.g., absence of TLS, vascular abnormalities, and lymph node metastasis), leading to T_PEX_ depletion (50–54, 68, 69); 2) Overwhelming differentiation pressure from chronic inflammation and antigen load, which exhausts the progenitor reservoir (37, 73); 3) Metabolic sabotage within the TME, impairing the mitochondrial fitness of T_PEX_ cells (39, 40). The consequence is a collapsed regenerative engine for antitumor immunity. The T cell compartment becomes dominated by terminally exhausted, dysfunctional T_PEX_ cells, incapable of controlling tumor growth. This failure to replenish effector cells leads to diminished immune pressure, allowing for unchecked tumor expansion, evolution of antigen-loss variants, and eventual metastatic dissemination.

Consequently, the frequency of T_PEX_ cells is a balance between supportive signals and differentiation pressures that deplete the pool by driving terminal exhaustion. The dynamic regulation of T_PEX_ cells is a core modulator of anti-tumor immunity. Therapeutic strategies that successfully maintain, expand, or restore T_PEX_ cells, such as ICB, microenvironment modulators, or adoptive transfer of stem-like T cells, essentially work by restoring this critical regenerative capacity, thereby shifting disease progression from advancement to control.

## 6 Key transcription factors and cytokines regulating stem-like T cells

**Figure 3:**
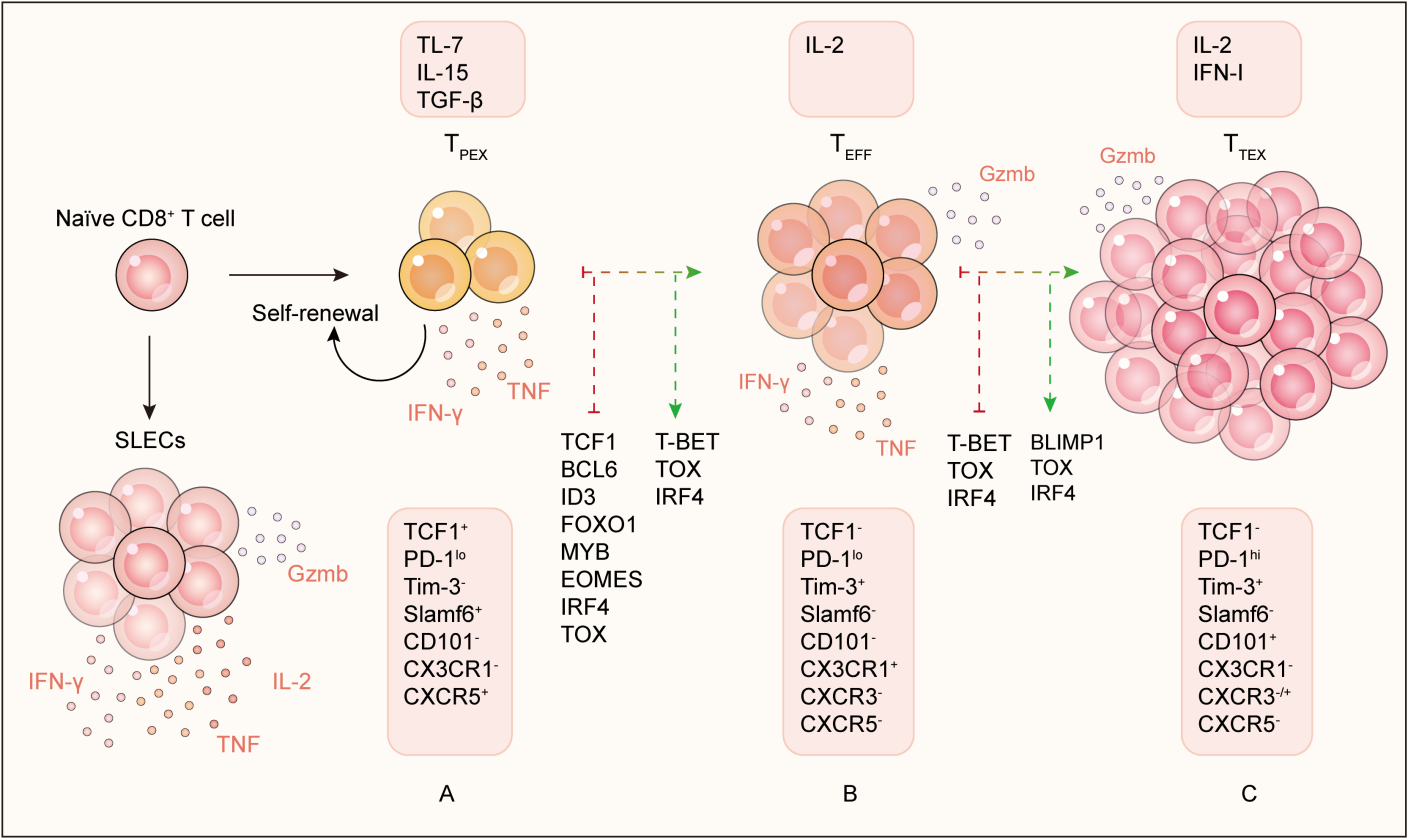
Key transcriptional factors and cytokines regulators. **(A)** The differentiation and function of T_PEX_ cells are controlled by TCF1, BCL6, EOMES, MYB, FOXO1, and ID3. These factors maintain stemness and prevent terminal differentiation. Cytokines such as TGF-β, IL-7, and IL-15 further support T_PEX_ cell survival and maintenance. **(B)** The differentiation T_EFF_ cell is dependent on T-BET, TOX, and IRF4. Cytokines such as IL-2 further support T_EFF_ cell development. **(C)** The differentiation of T_TEX_ cells is driven by BLIMP1, TOX, and IRF4. Chronic exposure to cytokines such as IFN-I and IL-2 can induce terminal exhaustion.

The maintenance and functional output of T_PEX_ cells are governed by a dynamic interplay of cell-intrinsic transcriptional programs and extrinsic signals from the TME. Here we summarized some key transcription factors (TFs) and cytokines, which can be broadly categorized into those controlling the overall exhaustion program, those maintaining stemness, and those promoting effector function and terminal differentiation (**Figure 3**), with detail available in **Table 3**.

**Table 3:** Key transcription factors and cytokines regulating stem-like T cells.

Role	Transcription factors and cytokines	Function	References
Stemness maintenance	TCF1	Maintain the stem cell characteristics of T_PEX_ cells, inhibit their differentiation into T_EFF_ cells, and promote self-renewal and long-term survival.	(22, 31, 74, 75, 84)
	BCL6	Promote the stem-like program of T_PEX_ cells, inhibit BLIMP1-mediated terminal differentiation, and maintain the persistence of T_PEX_ cells.	(75, 76, 84)
	FOXO1	Promote the formation, and maintain the long-term survival and stem cell characteristics of T_PEX_ cells.	(79– 81)
	IL-7/IL-15	Cooperate to govern T_PEX_ cell formation and homeostasis.	(102–106)
	TGF-β	Maintain the stem cell state of T_PEX_ cells by inhibiting their differentiation and promoting their residence in lymphoid tissues.	(111–114)
Differentiation	BLIMP1	Drive the T_PEX_ differentiation into exhausted T cells via promoting inhibitory receptor expression (e.g., PD-1) while directly repressing stemness genes like TCF1	(75, 90, 91)
	T-BET	Drives T_PEX_ cells differentiation and enhances the cytotoxicity of T_EFF_ cells.	(17, 92, 93)
	TOX	Highly expressed in T_TEX_ cells, and further promoting the exhausted programs transcriptionally and epigenetically.	(9, 75, 94–96)
	IL-2	Promotes the differentiation of T_PEX_ cells into effector T cells by inducing BLIMP1 expression, and excess IL-2 lead to T cell exhaustion.	(107)
Dual effect	IRF4	Promotes early effector differentiation, but under chronic stimulation it cooperates with TOX to repress TCF1 and enforce exhaustion	(18, 97)
	IL-10	(Mouse model) Promoted T cell exhaustion and impaired antitumor responses in mouse model; (Patients) PEGylated IL-10 treatment enhanced intratumoral CD8+ T cell expansion and effector function and was related to tumor regression.	Mouse model (39, 108); Patients (109, 110)
	IFN-I	Early stage: enhance T cell expansion; Late stage: promoted CD8^+^ T cell terminal exhaustion	(21, 115)

TPEX, Progenitor or precursor exhausted T; TEFF, Effector-like exhausted T; TTEX, Terminally differentiated exhausted T.

### 6.1 Transcriptional circuit for stemness maintenance

A strong consensus exists around a core set of transcription factors (TFs) that are necessary and instructive for the stemness and persistence of T_PEX_ cells, including TCF1 (22, 31, 74), BCL6 (75, 76), ID3 (77, 78), FOXO1 (79–81), and EOMES (82, 83). Among these TFs, TCF1 is the foremost and crucial for T_PEX_ cell formation and maintenance (22, 31, 74). TCF1 establishes the transcriptional network required for T_PEX_ cell differentiation by promoting EOMES and BCL6 expression, while suppressing T-BET and BLIMP1 expression (75, 84). Consequently, TCF1-deficient CD8^+^ T cells result in the impaired maintenance of T cells response and poor efficacy of ICB in mouse models (85). Similarly, the deletion of BCL6 displays reduced T_PEX_ cell abundance and attenuates the long-term tumor control (75, 76, 84). In contrast, overexpression of TCF1, ID3, or FOXO1 in CD8^+^ T cells or in CAR-T cells could improve the persistence and tumor control (75, 76, 79, 81, 86). Another key regulator, MYB, is typically expressed in hematopoietic stem cells and in human stem-like T cells. Evidence shows that MYB-deficient T cells fail to respond to ICB (87), and *Myb* overexpression in CD8^+^ T cell enhanced the T_PEX_ cell formation and improved tumor control in mouse models (88, 89).

### 6.2 Regulatory that drive differentiation and determine functional fate

If TCF1, BCL6, and FOXO1 form the “brakes” on differentiation, a separate set of factors act as the “accelerator”, pushing T_PEX_ cell toward effector and exhausted fates.

One such regulator is BLIMP1, which promotes inhibitory receptor expression (e.g., PD-1, LAG-3) while directly repressing stemness genes (*Tcf7, Bcl6, Ccr7, Sell, Cxcr5, Il-7r*) (75, 90); reciprocally, TCF1 also repressed BLIMP1 (91). Consequently, BLIMP1 deletion expands the T_PEX_ pool and improves responses to immunotherapy (75). Similarly, T-BET is essential for forming effector subsets and can antagonize the exhaustion marker PD-1 expression (17, 92, 93).

Moreover, persistent antigen signaling established a robust causal link to terminal exhaustion via the induction of TOX. TOX is highly expressed in T_TEX_ cells and necessary for the full exhausted phenotype (9, 94, 95). Its absence preserves TCF1 expression and stem-like potential of T_PEX_ cells (75, 96). IRF4 exhibits kinetic complexity: it promotes early effector differentiation but under chronic stimulation cooperates with TOX to repress TCF1 and enforce exhaustion (18, 97).

The roles of ID2 and EOMES appear context-dependent. ID2 expressed in T_TEX_ cells and generally antagonizes TCF1/BCL6 in chronic LCMV infection (98–100), yet its loss in tumor model impairs T_PEX_ maintenance and anti-PD-1 response (101). EOMES is highly expressed in T_PEX_ cells but its deficiency reduces T_TEX_ formation while expanding the T_PEX_ compartment (163), suggesting a complex, stage-specific function that warrants further investigation.

### 6.3 Cytokines as fate-switching signals

Beyond transcription factors, cytokines critically shape T_PEX_ cell fate, though their necessity within tumors *in vivo* is often nuanced and context-dependent.

Homeostatic cytokines IL-7 and IL-15 are established promoters of the stem-like state, cooperating to instruct T_PEX_ cell differentiation (102–104) and used in culture to generate stem-like CAR-T cells (105, 106). Conversely, IL-2 predominantly drives effector differentiation by inducing BLIMP1 expression; engineered IL-2Rβγ agonists synergize with PD-1 blockade by expanding the T_PEX_-derived effector pool (107). However, the role of IL-10 appears to be more complex. In a murine melanoma model, IL-10 signaling promoted T cell exhaustion and impaired antitumor responses (108). But PEGylated IL-10 treatment enhanced intratumoral CD8^+^ T cell expansion and effector function and was related to tumor regression in cancer patients (109, 110). Moreover, IL-10 administration metabolically reprograms T_TEX_ cells, enhancing antitumor immunity in mouse tumor models independently of T_PEX_ cell (39). Thus, further research is necessary to fully understand the precise effect of IL-10 on T_PEX_ cells.

The roles of TGF-β defy their traditional immunosuppressive labels. In tumor models, loss-of-function studies show TGF-β induced BCL6 expression in CD8^+^ T cells and was essential for maintaining the T_PEX_ pool by enforcing residency and limiting premature differentiation (111–113). Consistently, TGF-β treatment enhances the stemness of CAR-T cells and improve antitumor efficacy (114).

IFN-I promote T_TEX_ cells by antagonizing the formation and maintenance of T_PEX_ cells (21). In the TME, IFN-I and IFN-II contributed to T cell exhaustion and activated resistance programs in tumor cells that limit anti-tumor T cell responses (21, 115).

## 7 Implication of stem-like T cells in immunotherapy

Due to the capacity of intrinsic progenitor properties, including self-renewal and multilineage differentiation potential, T_PEX_ cells serve as the cornerstone of durable anti-tumor immunity and clinical response to immunotherapy (14, 35). Correlative preclinical and clinical evidence across multiple cancer types demonstrated that increased intratumoral T_PEX_ cell abundance predicts improved immunotherapy outcomes, including enhanced T cell persistence and objective response rates (20, 22, 30, 68, 69). Consequently, T_SL_ cells represent both a predictive biomarker for therapeutic efficacy and a promising target for next-generation immunotherapies (**Table 4**).

**Table 4:** Potential therapeutic strategies targeting stem-like cells.

Immunotherapy	Strategies to enhance the stemness of T_PEX_	References
ICB	Combined with radiotherapy or chemotherapy to enhances T_PEX_ cell priming and recruitment	(130, 131, 134)
	Combined with epigenetic reprogramming using DNMT inhibitors (such as azacytidine) or LSD1 inhibitors	(132, 133)
	Combined with metabolic interventions	(39)
ACT	Substitution of IL-2 with homeostatic γ-chain cytokines (e.g., IL-7, IL-15, or IL-21)	(136)
	The modulation of TCR signaling strength during activation (e.g., through altered peptide ligand or co-stimulation)	(9, 139)
	Engineering CAR-T cells with stem- and memory-like phenotypes	(81, 145–147)
Cancer vaccination	Targeting of common tumor antigens or individualized neoAg	(22, 150–152)
	Combined with ICB	(153–155)

ICB, Immune checkpoint blockade; ACT, Adoptive cell immunotherapy; T_PEX_, Progenitor or precursor exhausted T.

### 7.1 Immune checkpoint blockade

Immune checkpoints are actually a normal part of the immune system with the role of preventing immune response from being too strong to destroy healthy cells in the body. As described before, when persistent antigen stimulation, T cells will undergo an exhausted phenotype with the increased expression levels of inhibitory receptors (e.g., PD-1 and CTLA4) (19). Notably, although T_PEX_ cells share some features of exhaustion, they retain proliferative capacity, self-renewal, and lineage plasticity, acting as the progenitor population that continually replenishes the T_TEX_ cells compartment, and responses to checkpoint blockade. Current ICB targeting CTLA-4 and PD-1 receptors have received positive outcomes (19, 116) and truly revolutionized the treatment of cancer patients with melanoma (117), breast cancer (118), lung cancer (119), and other cancers (120, 121).

Crucially, the mechanistic basis of ICB efficacy has been refined through detailed dissection of the T cell compartment. Accumulating evidence have suggested that the clinical benefit of PD-1/PD-L1 blockade is driven predominantly by the expansion and differentiation of T_PEX_ cells, rather than the functional restoration of T_TEX_ cells (5, 9, 23). T_PEX_ cells, characterized by TCF1^+^ PD-1^+^ expression, retain self-renewal capacity and serve as a proliferative reservoir. In contrast, T_TEX_ cells exhibit a fixed epigenetic and transcriptional state characterized by chromatin remodeling, TOX-driven transcriptional reprogramming, and metabolic exhaustion, rendering them refractory to reinvigoration (9, 122–124).

Preclinical studies established that T_PEX_ cells serve as the primary reservoir for tumor-specific T cell upon PD-1/PD-L1 inhibitors (36, 59). In murine models of chronic LCMV infection and tumors, PD-1 inhibitor induces the proliferation and differentiation of T_PEX_ subset into T_EFF_ cells, driving tumor control (11, 23). Clinically, the association between T_PEX_ cells and response to ICB is context-dependent and continues to be refined. Seminal work in melanoma demonstrated that higher frequencies of intratumoral T_PEX_ cells correlate with prolonged progression-free survival (PFS) and objective response to anti-PD-1 therapy, and that responding patients exhibit clonal expansion of these cells, replenishing the cytotoxic T cell pool post-treatment (22, 125, 126). These findings established T_PEX_ cells as a promising biomarker in this immunogenic cancer. However, subsequent studies across diverse tumor types and patient cohorts have revealed a more nuanced picture. While some reports corroborate a positive association with clinical benefit, others find correlations primarily with PFS rather than with objective response rates per se, and in certain contexts, the abundance of T_PEX_ cells alone does not robustly predict clinical outcomes (15, 49, 59, 127–129). These discrepancies may stem from differences in tumor immunogenicity, prior therapies, T cell sampling site (e.g., blood vs. tumor), and the precise phenotypic definition of the stem-like population. Therefore, while T_PEX_ cells are mechanistically crucial for sustaining antitumor immunity, their utility as a universal predictive biomarker requires further validation in specific cancer types and treatment settings.

Currently, rational combination therapies targeting T_PEX_ cell amplification have shown mechanistic and clinical synergies. Firstly, radiotherapy promotes immunogenic cell death, releasing DAMPs that activate dendritic cells by the cGAS-STING pathway. This cascade enhances T_PEX_ cell priming and recruitment to the tumor site and, when used in combination with anti-CTLA-4, amplifies the distal response (130, 131). Secondly, epigenetic reprogramming using DNMT inhibitors (such as azacytidine) or LSD1 inhibitors reverses T cell exhaustion by demethylating the *Tcf7* enhancer. This sustains T_PEX_ to a stem-cell-like state, restoring anti-PD-1 reactivity in preclinical models (132, 133). Thirdly, chemotherapy (such as oxaliplatin) upregulates CXCL10 in tumor vessels via IFN-γ signaling. The resulting chemokine gradient drives T_PEX_ cell homing into tumors, enhancing the efficacy of ICB (134).

### 7.2 Adoptive cell immunotherapy

Studies on adoptive cell therapy (ACT) demonstrate that sustained antitumor immunity depends critically on the persistence of reinfused cells rather than their immediate cytotoxic capacity upon transfer because prolonged or excessive stimulation during T cell expansion is known to promote exhaustion and can compromise the functional potency of adoptively transferred cells (135). Consequently, contemporary ACT protocols prioritize generating less differentiated cell subsets over terminally differentiated populations to maximize antitumor efficacy in this context. Key strategies focus on culturing conditions that favor progenitor-like states. A pivotal approach involves the substitution of IL-2 with homeostatic γ-chain cytokines (e.g., IL-7, IL-15, or IL-21) during *ex vivo* expansion (136). Unlike IL-2, which can drive terminal effector differentiation and exhaustion, these cytokines promote homeostatic proliferation and help maintain or upregulate stem/progenitor-associated genes (such as *Tcf7* and BCL6), thereby preserving a less differentiated, more persistent T cell product (103, 137, 138). Separately, the modulation of TCR signaling strength during activation (e.g., through altered peptide ligand or co-stimulation) is another critical lever to prevent over-stimulation and exhaustion, working in concert with cytokine conditioning to optimize T cell quality (9, 139). Additional strategies include augmenting stemness-promoting pathways like Notch signaling (140) and inhibiting transcription factors linked to terminal dysfunction (e.g., BLIMP1) (75).

Exogenous T cell therapies, particularly CAR-T immunotherapy, have established a new standard of care for several relapsed or refractory B-cell malignancies, including certain types of large B-cell lymphoma and B-cell acute lymphoblastic leukemia (141–144). Their application is actively being explored and is expanding into other well-defined hematologic and solid tumor settings. Notably, pre-infusion products enriched in CAR-T_TEX_ cell populations correlate with inferior outcomes, whereas stem- and memory-like phenotypes associate with higher response rates (145, 146). Although comprehensive clinical characterization of T_PEX_ phenotypes in CAR-T products remains limited, recent work identified PD-1^+^ TCF1^+^ CAR-T_PEX_ cells as predictors of improved clinical outcomes (147). Preclinically, engineered CAR-T models overexpressing T_PEX_-associated TFs exhibit enhanced stem-like phenotypes, expansion potential, persistence, and therapeutic efficacy (81). Similarly, pre-existing TLS or APC-dense niches may be essential for generating and sustaining CAR-T_PEX_ phenotypes; thus, fostering these microenvironments may augment their persistence (2, 148). Furthermore, utilizing T_PEX_ cells and their molecular signatures as predictive biomarkers may optimize CAR-T clinical management.

Collectively, these findings underscore the paramount importance of preserving and augmenting T_PEX_ cells to enhance persistence and therapeutic outcomes in ACT, particularly in CAR-T immunotherapy.

### 7.3 Cancer vaccination

Therapeutic vaccination targeting either shared tumor antigens or patient-specific neoantigen (neoAg) pools represents a promising strategy to activate antitumor T cell immunity (149, 150). Leading vaccination approaches aim to harness the self-renewal capacity, long-term persistence, and multilineage differentiation function of T_PEX_ cells through targeting of common tumor antigens or individualized neoAg (150, 151). Preclinical studies demonstrate that the efficacy of therapeutic vaccination depends critically on T_PEX_ cells; thus, enriching these populations during vaccination could theoretically enhance antitumor responses (22, 152). While vaccines initiate *de novo* T cell responses against tumors, functional exhaustion may limit their activity. Consequently, many clinical vaccine trials employ combinatorial approaches with ICB (153).

Accordingly, vaccines specifically designed to induce T_PEX_ cell populations have been developed and show potent synergy with ICB in tumor models (154, 155). Although clinical outcomes from tumor vaccine trials have yielded inconsistent results, expanding T_PEX_ cells represents a key consideration for improving future vaccine efficacy.

### 7.4 Clinical translation: challenges and future directions

While the pivotal role of T_PEX_ cells in immunotherapy efficacy is well-established preclinically, translating these insights into clinical practice faces several challenges and opportunities.

#### 7.4.1 Operationalizing stem-like T cells as predictive biomarkers

The development of T_PEX_ cell abundance as a clinically useful biomarker requires standardized and feasible measurement protocols. Critical considerations include sampling source, assay methodology, and temporal dynamics.

**Tissue-based assessment:** Direct measurement in the TME via multiplex immunofluorescence (e.g., co-detection of TCF1^+^ PD-1^+^ TIM-3^−^ cells in FFPE samples), scRNA seq, or spatial transcriptomics provides the most relevant data. Clinical studies across melanoma (20, 22), HNSCC (156), and hepatocellular carcinoma (128) have correlated higher intratumoral T_PEX_ cell frequencies with improved PFS following ICB treatment. However, T_PEX_ cell abundance alone may be insufficient as a prognostic marker of therapy response because responsiveness may require the presence of both T_PEX_ cells and niches permissive for their differentiation, such as TLS and APC niche. Consistent with this idea, T_PEX_ cell supportive niches are enriched in tumors of patients with beneficial therapeutic responses (15, 49, 127–129).

**Blood-based monitoring:** Peripheral blood analysis offers a minimally invasive alternative for dynamic monitoring. Preclinical studies indicate T_PEX_ cells are activated during the early phase of antitumor immune responses and predominantly reside in the TDLN, and these T cells subsequently traffic into the TME via tumor-associated high endothelial cells to exert their function (2, 65, 157). Several clinical studies have shown that peripheral T cell expansion predicts tumor infiltration and clinical response (158, 159). Their expansion in circulation has been observed following combination therapies [e.g., CD122-directed IL-2 with radiotherapy/anti-PD1 (72)]. In patients with advanced NSCLC, a higher frequency of circulating T_PEX_ cells was associated with improved survival (160). Techniques such as multiplexed flow cytometry and TCR sequencing enable tracking of these populations over time.

**Timing of assessment:** Biomarker utility likely depends on the timepoint of evaluation. The examination of T_PEX_ cells may be most informative at baseline (predictive of response) and early during treatment (pharmacodynamic indicator of T_PEX_ cell expansion). Post-treatment sampling could inform the durability of response. Overall, it is a critical need for longitudinal tracking through serial sampling (tissue or blood), which is essential to understand clonal dynamics and functional evolution throughout treatment and disease progression.

#### 7.4.2 Implications for treatment sequencing and rational combinations

The central role of T_PEX_ cells informs rational therapeutic design. Treatment sequencing is critical. Modalities designed to expand or generate T_PEX_ pools (e.g., certain vaccines or epigenetic modulators) could be deployed prior to or alongside ICB to “prime” the responsive reservoir. Conversely, for patients progressing on ICIs, strategies to replenish the T_PEX_ compartment (e.g., ACT with stem-like phenotypes) may be necessary.

In addition, given the complexity of sustaining an effective T cell response, combination strategies targeting multiple nodes are promising and more likely to yield durable benefits. These include combining ICB to initiate T_PEX_ proliferation with: agents that foster supportive niches (e.g., VEGF inhibitors for vascular normalization), epigenetic modulators to reinforce stemness programs (29), engineering approaches (e.g., next-generation CAR-T designs) to confer exhaustion resistance (161, 162), metabolic interventions to enhance mitochondrial fitness (39), and so on. Notably, T_PEX_ profiling before and during treatment could guide patient selection, ensuring that these combination strategies are applied to individuals most likely to benefit.

#### 7.4.3 Caveats in extrapolating from murine models to human cancers

While indispensable for mechanistic discovery, key limitations exist when extrapolating from murine models to human cancers. Laboratory models often employ defined antigens and rapid tumor growth, potentially oversimplifying the chronicity and antigen heterogeneity characteristic of human disease. Furthermore, the human TME exhibits greater cellular and spatial complexity, and the endogenous T cell repertoire is far more diverse than the restricted repertoires typical in mouse studies.

Therefore, while murine models robustly elucidate fundamental principles, quantitative predictions (e.g., required T_PEX_ frequency for response) and therapeutic efficacy of specific interventions must be rigorously validated in human clinical trials and through studies using patient-derived models.

## 8 Conclusions

In conclusion, T_PEX_ cells are conclusively demonstrated as central mediators of antitumor immunotherapies, for their progenitor-like properties of self-renewal, differentiation plasticity, and long-term persistence. Their presence critically determines therapeutic outcomes across ICB, ACT, and cancer vaccination. Future research may prioritize strategies preserving T_PEX_ functionality, engineering supportive microenvironments, and leveraging T_PEX_-associated signatures for biomarker development. Ultimately, targeting T_PEX_ cells represents a promising paradigm shift to overcome immunotherapy resistance and achieve sustained clinical responses.

## Glossary

ACT: Adoptive cell therapy
APC: Antigen-presenting cell
BCL: B-cell lymphoma
Blimp1: B-lymphocyte-induced maturation protein 1
CAR-T: Chimeric Antigen Receptor T-Cell Immunotherapy
CCL: C-C chemokine ligand
CCR: C-C chemokine receptor 7
cGAS–STING: Cyclic GMP-AMP synthase–Stimulator of interferon genes pathway
CXCL: C-X-C chemokine ligand
CXCR: C-X-C chemokine receptor
DAMPs: Damage-associated molecular patterns
DNMT: DNA methyltransferases
FAO: fatty acid oxidation
FOXO1: Forkhead box O1
GZMB: Granzyme B
HK2: hexokinase 2
HNSCC: Head and neck squamous-cell carcinoma
ICB: Immune checkpoint blockade
IFN: Interferon
IL: Interleukin
Il7rα: Interleukin 7 receptor subunit alpha
IRF: Interferon regulatory factor
LAG3: Lymphocyte-activation gene 3
LCMV: Lymphocytic choriomeningitis virus
LEF1: lymphoid enhancer binding factor 1
LSD1: Lysine Specific Demethylase 1
MPECs: Memory precursor effector cells
mDCs: Migratory dendritic cells
NFAT: Nuclear Factor of Activated T-Cells
NSCLC: Non-small-cell lung cancer
OXPHOS: Oxidative phosphorylation
PFS: Progression-free survival
S1P: Sphingosine-1-phosphate
scRNA-seq: Single-cell RNA sequencing
Sell: L-selectin
SLECs: Short-lived effector cells
SRC: Spare respiratory capacity
STAT5: Signal Transducer and Activator of Transcription 5
T-bet: T box expressed in T cell
TCF1: Transcription factor 1
TCR: T cell receptor
TCZ: T cell zone
TDLN: Tumor-draining lymph nodes
T_EEF_: Effector-like exhausted T
TIM3: T cell immunoglobulin and mucin domain-containing protein3
T_INT_: Intermediate exhausted T
TLS: Tertiary lymphoid structures
TME: Tumor microenvironment
T_PEX_: Progenitor or precursor of exhausted T
T_SL_: Stem-like T
T_TEX_: Terminally differentiated exhausted T
